# Effects of (±) 3,4-Methylenedioxymethamphetamine (MDMA) on Sleep and Circadian Rhythms

**DOI:** 10.1100/tsw.2007.214

**Published:** 2007-11-02

**Authors:** Una D. McCann, George A. Ricaurte

**Affiliations:** Departments of Psychiatry and Behavioral Sciences and Neurology, The Johns Hopkins School of Medicine, Johns Hopkins Bayview Medical Center, 5501 Hopkins Bayview Drive, Baltimore, MD 21224, USA

**Keywords:** neurotoxicity, serotonin (5-hydroxytryptamine, 5-HT), drug abuse, MDMA (Ecstasy)

## Abstract

Abuse of stimulant drugs invariably leads to a disruption in sleep-wake patterns by virtue of the arousing and sleep-preventing effects of these drugs. Certain stimulants, such as 3,4-methylenedioxymethamphetamine (MDMA), may also have the potential to produce persistent alterations in circadian regulation and sleep because they can be neurotoxic toward brain monoaminergic neurons involved in normal sleep regulation. In particular, MDMA has been found to damage brain serotonin (5-HT) neurons in a variety of animal species, including nonhuman primates, with growing evidence that humans are also susceptible to MDMA-induced brain 5-HT neurotoxicity. 5-HT is an important modulator of sleep and circadian rhythms and, therefore, individuals who sustain MDMA-induced 5-HT neurotoxicity may be at risk for developing chronic abnormalities in sleep and circadian patterns. In turn, such abnormalities could play a significant role in other alterations reported in abstinent in MDMA users (e.g., memory disturbance). This paper will review preclinical and clinical studies that have explored the effects of prior MDMA exposure on sleep, circadian activity, and the circadian pacemaker, and will highlight current gaps in knowledge and suggest areas for future research.

## STIMULANT DRUG ABUSE, NEUROTOXICITY, AND SLEEP

Psychostimulants, by definition, increase arousal and suppress sleep. A consequence of stimulant abuse, therefore, is a disruption in normal sleep-wake patterns. For example, during binges of cocaine or methamphetamine, users sometimes forego sleep for days[1]. Cessation of drug use, whether because of exhaustion or deliberate drug cessation, is invariably associated with several days of excessive sleepiness and/or hypersomnolence[2], with one study demonstrating continued sleep disruption for weeks[3]. Although the neural substrates underlying stimulant-induced arousal and sleep-wake disturbances following stimulant binges are not fully understood, they are known, at least in part, to involve brain dopamine and norepinephrine systems that modulate normal arousal states[4].

In addition to disturbances in sleep and circadian rhythms that are due to acute and subacute stimulant effects of drugs via pharmacologic actions of drugs, certain stimulant drugs, such as 3,4-methylenedioxymethamphetamine (MDMA), may produce more lasting disruptions to sleep and circadian activity because they have neurotoxic potential toward brain monoaminergic neurons known to modulate normal sleep-wake patterns[5]. In particular, the potential for MDMA to produce a lasting loss of brain serotonin (5-HT) axonal markers in animals was first noted more than 2 decades ago[6,7,8,9]. Since these early findings in rodents, evidence for MDMA-induced brain 5-HT neurotoxicity has been confirmed and extended in numerous laboratories[10,11,12] and in numerous animal species, including guinea pigs[8]; mice[13]; squirrel, cynomolgus, and rhesus monkeys[14,15,16,17,18]; and baboons[19]. In nonhuman primates, 5-HT innervation can remain abnormal for years after MDMA administration[20].

A growing body of data suggests that humans who use MDMA as a recreational drug can also sustain 5-HT neurotoxicity. Early studies demonstrated that MDMA users, like MDMA-treated monkeys with documented serotonergic injury[21], have selective deficits in cerebrospinal fluid measures of 5-hydroxyindoleacetic acid (5-HIAA), the major metabolite of 5-HT[22]. Later studies using neuroimaging methods with positron emission tomography (PET) and single photon emission computed tomography (SPECT), and radioligands that bind to the 5-HT transporter, revealed that MDMA users, compared to non-MDMA–using controls, have reductions in 5-HT transporter density[23,24,25,26,27,28]. Similar reductions in 5-HT transporter density are seen in baboons with documented MDMA-induced brain 5-HT injury[19,29].

Although the functional consequences of MDMA-induced 5-HT neurotoxicity are not clear, there have been reports of abstinent MDMA users that developed disorders of mood, anxiety, cognition, impulsivity, and sleep following MDMA exposure[30,31,32,33]. The most consistently observed functional deficit in abstinent MDMA users has been a problem of cognitive function, possibly because it has been the area that has received the greatest attention[33]. Notably, serotonin is known to play a role in sleep and circadian activity[5,34] cognitive function[35], endocrine regulation[36], and impulsivity[37,38], raising the possibility that MDMA users with disorders in these behavioral spheres are experiencing symptoms as a result of MDMA-induced brain 5-HT injury. However, alterations in sleep and circadian rhythm modulation alone can lead to alterations in cognitive function, mood, impulsivity, and endocrine function, creating the possibility that alterations in the sleep-wake cycle could play a role in other abnormalities seen in abstinent MDMA users. After a brief discussion on the role of brain 5-HT systems and sleep, this paper will review the available preclinical and clinical literature that has evaluated the effects of MDMA exposure on sleep and circadian activity, and will discuss areas of future research interest.

## SEROTONIN AND SLEEP

Brain 5-HT neurons play an important role in sleep, through their neuromodulatory actions[5,39], their influence on the endogenous circadian pacemaker[34,40,41], and through their actions on muscles involved in airway patency during sleep[42,43]. Therefore, humans who sustain brain 5-HT injury as a result of their MDMA use may be at risk for developing altered sleep architecture, disruptions in circadian rhythms, and/or sleep-disordered breathing.

## EFFECTS OF MDMA ON SLEEP: PRECLINICAL STUDIES

### Acute and Persistent Effects of MDMA on Circadian Activity

A single study investigated the effects of MDMA on circadian activity patterns. Balogh and colleagues[44] evaluated the effects of single acute doses of systemically administered MDMA on wakefulness, activity, circadian patterns, brain glucose utilization, and [$^3$H] paroxetine binding in Dark Agouti rats. As might be anticipated from an amphetamine analog, activity and wakefulness increased for at least 6 h after MDMA administration in drug-naïve animals and also in animals that had received a previous single dose of systemically administered MDMA 3 weeks earlier. Circadian patterns of motor activity and sleep were altered for approximately 5 days after drug treatment, and alterations in gross motor activity, wakefulness, and "deep" slow-wave sleep were still evident at the last data collection point, a full month after MDMA administration. In addition to changes in activity, sleep, and circadian patterns, MDMA administration led to acute global increases in cerebral glucose utilization, with reduced glucose utilization in the same brain regions 3 weeks later. Animals also showed significant reductions in cortical, but not striatal, [$^1$H] paroxetine binding to the 5-HT transporter measured 3 weeks after drug administration.

## MDMA-Induced Changes in Circadian Clock Function

A series of studies by Biello and colleagues[40,45,46,47] explored the acute and longer-term effects of MDMA on the ability of mammals to re-entrain the circadian clock. In mammals, the circadian clock is located in the suprachiasmatic nuclei (SCN) of the hypothalamus[48], and receives inputs from the retina, the lateral geniculate nucleus, and the serotonergic neurons within the raphe nuclei[34,40,41]. A variety of stimuli have been shown to "re-entrain" the circadian clock, including light via the retinohypothalamic tract[49], 5-HT agonists[50,51], and triazolam[52]. It has been established that the circadian clock is modulated by brain 5-HT[34,50], and the studies by Biello and colleagues tested the hypothesis that MDMA, by virtue of its 5-HT neurotoxic properties, would lead to alterations in the ability of the circadian clock to respond normally to various stimuli..

Their first study in rats[45] tested the ability of MDMA to alter the ability of the circadian clock to reset in response to the 5-$HT_1$/5$HT_7$ receptor agonist 8-hydroxy-2-(dipropylamino)tetralin (8-OH-DPAT). Using an *in vitro* slice method and pharmacological stimulation with 8-OH-DPAT, the authors measured electrical responses of sections from the SCN of animals that had been treated with MDMA 6–10 days and 20 weeks previously. Phase advances to 8-OH-DPAT in MDMA-treated rats were attenuated in both groups of animals, indicating a long-lasting alteration in circadian clock function.

In a second study, Colbron and colleagues[46] used a within-animal design and *in vivo* circadian activity monitoring (wheel running) in Syrian Hamsters before and after treatment with MDMA or saline. Both treatment groups were exposed to sequential stimulation with 8-OH-DPAT and a 15-min light pulse before and 3–5 weeks after MDMA administration. Their results indicated that MDMA treatment attenuated the ability for animals to phase shift in response to both 8-OH-DPAT and light.

In their third study of MDMA's disrupting effects on circadian clock function[47], the role of brain dopamine and temperature in MDMA-induced circadian clock alterations was assessed in male Wistar rats. In particular, doses of the catecholamine synthesis inhibitor AMPT (alpha-methyl-para-tyrosine) or the dopamine-2 receptor blocker haloperidol, which had previously been shown to prevent MDMA-induced temperature increases, were coadministered with MDMA. Using the same *in vitro* slice method of the SCN as employed previously[45], electrical responses of the SCN to 8-OH-DPAT were measured and were compared to those from animals that had been treated with MDMA alone, AMPT alone, haloperidol alone, or saline. As expected, both AMPT and haloperidol prevented the acute hyperthermic effect of MDMA. However, neither treatment prevented the effects of MDMA on 8-OH-DPAT–induced phase shifts in SCN firing. Prior treatment with neither haloperidol alone nor AMPT alone influenced 8-OH-DPAT–induced phase shifts.

In their most recent study[40], the ability of previous MDMA treatment to alter the effects of triazolam-induced shifts in the circadian clock and the ability to re-entrain to a new light-dark cycle were evaluated in male Syrian hamsters *in vivo*, as measured by circadian activity patterns (wheel running). At the conclusion of the experiment, immunocytochemical methods were employed to assess the status of 5-HT axon terminals in the SCN. Animals previously treated with MDMA were found to have significantly reduced triazolam-induced phase shifts and to take longer to re-entrain to a new light-dark cycle. In the same animals, there was a significant reduction in 5-HT positive fibers in the SCN, leading the authors to conclude that MDMA-induced toxicity toward 5-HT axon terminals in the SCN led to impairments in photic and nonphotic responses of the circadian clock.

Taken together, data from rodents previously treated with MDMA are highly consistent in their conclusions. In particular, MDMA treatment has been shown to lead to protracted alterations in circadian activity patterns and, in addition, leads to dysfunction of the circadian pacemaker in the SCN. It is not yet known whether these protracted effects of MDMA on the sleep-wake cycle generalize to all species, and whether abnormalities in sleep and circadian rhythms lead to altered psychomotor performance, altered circadian endocrine function, or changes in other behavioral domains.

## EFFECTS OF MDMA ON SLEEP: CLINICAL STUDIES

Four studies collected objective measures of sleep and alertness in MDMA users[53,54,55,56]. Of these, one[55] measured the acute effects of nocturnal MDMA on vigilance, psychomotor performance, and impulsivity during a night of sleep deprivation, whereas the other three evaluated the sleep EEG in abstinent MDMA users. These will be discussed, in turn.

Kuypers and colleagues studied 14 healthy individuals who had previous experience with MDMA and who underwent two nights of sleep deprivation separated by at least 7 days[55]. Subjects received either placebo or oral MDMA (125 mg in two split doses separated by 4 h), and underwent repeated cognitive testing during each night. Although subjects developed decreased vigilance with prolonged wakefulness on both study nights, MDMA attenuated this effect compared to placebo. Similarly, self-rated sleepiness scales were lower following MDMA than following placebo. Following MDMA, subjects demonstrated impairments in tracking and divided attention tasks that were worse than those seen following placebo. These observations are consistent with a psychostimulant effect of MDMA and also suggest that nocturnal administration of MDMA may lead to performance impairments that are additive to those produced by sleep deprivation.

In the first all-night sleep polysomnographic study of MDMA users, Allen and colleagues[53] compared sleep EEGs in 23 abstinent MDMA users to those of 22 age- and gender-matched control subjects. MDMA users were found to have reductions in total sleep and non-REM sleep, with the latter primarily secondary to reductions in stage 2 sleep. Although the methods used did not permit conclusions regarding the relationship between MDMA-induced 5-HT neurotoxicity and alterations in sleep architecture, they were interpreted as being consistent with the notion that MDMA use can lead to persistent changes in sleep, possibly secondary to lasting functional changes in 5-HT neurons.

In a follow-up study, Ricaurte and McCann conducted all-night sleep studies in 25 abstinent MDMA users and 25 age- and gender-matched controls, before and after pharmacological challenge with the mixed 5-HT agonist m-chlorophenylpiperazine (m-CPP)[54]. MDMA users, as before, were found to have significant differences in sleep architecture compared to controls, but the nature of these differences were not identical to those found in the first study. MDMA users were also found to respond differently to m-CPP when compared to controls. In particular, although MDMA users had decreased stage 2 at baseline when to compared to controls at baseline, this difference did not reach statistical significance. On the other hand, MDMA users had significant increases in stages 3/4 (slow-wave sleep) compared to controls. Following m-CPP, stage 4 increased in MDMA users, but decreased in control subjects. In addition to differences between the two groups with regard to sleep stages, MDMA users were noted to have greater sleep efficiency than control subjects. Although the reasons for differences between the two studies was not clear, it was hypothesized that it might be related to differences in the amount of drug used in the two different MDMA cohorts as well as differences in the duration of abstinence from MDMA. Nevertheless, the observation that MDMA users had alterations in sleep architecture and a differential sleep response to a drug known to act on brain 5-HT neurons was viewed as consistent with the notion that MDMA-induced neurotoxicity leads to alterations in sleep.

In the most recent sleep study in abstinent MDMA users[56], sleep and cognitive function were assessed in 25 abstinent MDMA users and 23 non-MDMA–using controls before and after pharmacological challenge with the catecholamine synthesis inhibitor AMPT. The rationale was that both sleep and cognitive function are dually modulated by 5-HT and catecholamines, and that MDMA users, if they had sustained MDMA-induced 5-HT neurotoxicity, would be differentially effected by AMPT. Baseline sleep architecture was also altered in abstinent MDMA users compared to controls, and AMPT produced differential effects in MDMA users compared to controls on several sleep measures. In particular, at baseline, there were significant differences between MDMA users and controls, with MDMA users having less stage 2 sleep and more stage 1 sleep than controls. There were also trends suggesting decreased total sleep in MDMA users. These findings are quite similar to those found in the first study of sleep by this group[53]. Differential effects of AMPT were observed on measures of sleep latency and REM latency. In particular, following AMPT, there were greater increases in sleep latency in controls than in MDMA users, and there was increased REM latency in MDMA users, but decreased REM latency in controls. The authors then explored the relationship between MDMA user parameters and alterations in baseline sleep measures. Consistent with the hypothesis that exposure to MDMA was related to reductions in stage 2 sleep, significant negative correlations were found between time spent in stage 2 and total lifetime dose of MDMA and duration of MDMA use. Conversely, as would be predicted if increases in stage 1 were related to MDMA use, positive correlations were found between time spent in stage 1 and total lifetime dose of MDMA as well as duration of MDMA use. Taken together, these findings are supportive of the notion that MDMA-induced changes in 5-HT function lead to alterations in sleep architecture.

Limitations in studies of human MDMA users include that fact that most MDMA users have experience with other psychoactive substances, that drug use histories are retrospective, and the possibility that alterations in sleep and circadian rhythm could have predated MDMA use. However, as noted above, the relationship between markers of brain 5-HT axon integrity and alterations in sleep is certainly suggestive of a link between MDMA use and sleep/circadian rhythm abnormalities, since other commonly abused drugs (marijuana, cocaine, opiates, amphetamine) do not lead to a loss of brain 5-HT axonal markers. Further, parallel findings in animals that have not been exposed to other drugs provide a strong neurobiological foundation for the notion the MDMA has 5-HT neurotoxic potential and that MDMA-induced neurotoxicity leads to prolonged alterations in sleep, circadian rhythms, and circadian pacemaker function.

## CONCLUSIONS, KNOWLEDGE GAPS, AND FUTURE DIRECTIONS

Althought limited, the existing preclinical and clinical data regarding the long-term effects of MDMA on sleep and circadian rhythms are compelling. In particular, the existing animal data consistently demonstrate that MDMA leads to lasting alterations in circadian activity as well as dysregulation of the circadian clock in the SCN. Similarly, although studies in humans are more limited, all studies that have evaluated sleep in abstinent MDMA users using objective measures have found differences between MDMA users and controls, though the nature of these differences has not always been the same. The finding that alterations in sleep architecture are correlated with the extent of previous MDMA use, when considered with preclinical studies, suggests that MDMA use plays a role. It remains to be determined conclusively whether sleep disturbances in MDMA users are primarily due to MDMA-induced brain 5-HT neurotoxicity, or whether some components of sleep alterations in MDMA users are related to brain changes seen in abusers of other non-neurotoxic psychostimulants, such as cocaine[3].

There are a number of obvious gaps in our knowledge of MDMA’s effects on sleep and circadian regulation. First, there is a single study on the acute and subacute effects of MDMA on the sleep EEG in animals, and these findings should be extended to other animal species and for a longer duration post-MDMA administration. Such studies, if accompanied by parallel neurochemical and anatomical studies of the brain 5-HT system, will provide a basis on which to interpret sleep studies in abstinent human MDMA users. Similarly, the relationship between sleep abnormalities in MDMA-treated animals and alterations in behavioral spheres in the same animals (cognitive function, impulsivity, endocrine function, vigilance) would shed light on similar relationships in humans.

Future clinical studies of sleep in human MDMA users should attempt to address the relationship between lasting decrements in 5-HT axonal markers and sleep (e.g., by obtaining neuroimaging studies of the 5-HT system in the same individuals who undergo sleep studies), as well as the relationship between sleep abnormalities and behavioral changes seen in MDMA users (e.g., cognitive dysfunction, endocrine dysfunction, impulsivity, mood disorder). Studies comparing and contrasting sleep disturbances in MDMA users to those of abusers of other drugs of abuse known to produce lasting sleep alterations (e.g., cocaine, alcohol) could also be useful in differentiating the consequences of 5-HT neurotoxicity from the consequences of drug abuse *per se*. Use of more sophisticated and finely tuned methods, such as spectral analysis, could reveal subtle differences in the sleep EEG of MDMA users. Future sleep studies in MDMA users should include an assessment of breathing patterns to determine whether MDMA users have a higher incidence of sleep-disordered breathing than control subjects, because of altered 5-HT innervation to muscles involved in airway patency during sleep. Finally, efforts are needed to assess the possibility that the circadian clock is dysfunctional in abstinent MDMA users, as it is in animals previously treated with MDMA. Together, such studies would be of value, not only because they would provide a better understanding of the functional consequences of MDMA use, but because they will shed light on the role of brain 5-HT systems in normal sleep and circadian pattern regulation.

## ACKNOWLEDGMENTS

This work was supported by PHS Grants Hl071501 (McCann); DA05938, DA05707, and DA01796401 (Ricaurte); and NCRR grant M01RR002719 (Ford).
